# Heterologous Sequential mRNA Vaccination of Indian Rhesus Macaques Elicits Broad Binding and Neutralizing Antibody Responses Against Diverse Henipaviruses

**DOI:** 10.3390/v18050487

**Published:** 2026-04-23

**Authors:** Thomas B. Voigt, Noor Ghosh, Brandon C. Rosen, Taylor Newbolt, Johan J. Louw, Aaron Yrizarry-Medina, Christakis Panayiotou, Jack T. Mauter, Giovana de Figueiredo Godoy, Joshua Terao, Eva G. Rakasz, Matthew R. Reynolds, Dawn M. Dudley, David I. Watkins, Michael J. Ricciardi

**Affiliations:** 1 Department of Pathology, George Washington University School of Medicine and Health Sciences, Washington, DC 20037, USA; 2 Mabloc LLC, Washington, DC 20052, USA; 3 Medical Scientist Training Program, University of Miami Miller School of Medicine, Miami, FL 33136, USA; 4 Wisconsin National Primate Research Center, University of Wisconsin-Madison, Madison, WI 53726, USA

**Keywords:** Henipavirus (HNV), Nipah virus (NiV), Hendra virus (HeV), Langya virus (LayV), Mojiang virus (MojV), Ghana virus (GhV), antibodies, broad-spectrum vaccines

## Abstract

Henipaviruses (HNVs), including Nipah virus (NiV) and Hendra virus (HeV), are highly pathogenic and often lethal zoonotic viruses with broad species tropism and no approved human vaccines. The emergence of genetically divergent HNVs—including Ghana virus (GhV), Langya virus (LayV), and Mojiang virus (MojV)—emphasizes the need for broadly protective countermeasures. Here, we evaluated the antibody (Ab) responses to sequential mRNA vaccines encoding the membrane-bound attachment glycoprotein (gG) from NiV, GhV, and/or LayV in a pilot study with Indian rhesus macaques. Serum binding Ab responses were quantified by ELISA against five soluble gG antigens (NiV, HeV, GhV, LayV, MojV). Functional activity was assessed by neutralization assays using NiV, HeV, and GhV pseudoviruses, and by receptor-blocking ELISA. Sequential vaccination induced high-titer IgG binding against all five HNV gGs with increasing breadth after each dose. Pan-genus regimens elicited moderate neutralizing Ab titers against NiV, HeV, and GhV, whereas the NiV-only regimen elicited potent but narrow neutralization against NiV and HeV. Conversely, the GhV-LayV-GhV regimen elicited strong binding to GhV, LayV, and MojV gG and robust neutralization of GhV pseudovirus, but limited cross-reactivity to NiV and HeV. In this pilot study, we demonstrated that mRNA vaccination can elicit broadly reactive binding and neutralizing Ab responses across phylogenetically distant HNVs. Additionally, we show GhV pseudovirus neutralization for the first time. Collectively, these data provide a foundation for the development of next-generation pan-genus HNV vaccines capable of mitigating future HNV outbreaks.

## 1. Introduction

The Henipavirus (HNV) genus of viruses represents an ongoing and increasing threat to public health. HNV infection typically presents as a flu-like illness before progressing to more severe and oftentimes fatal symptoms that include respiratory and neurological involvement, including pulmonary edema and encephalitis [1,2]. Though HNV cases in humans total less than 1,000 to date, outbreaks of Nipah (NiV) and Hendra (HeV) viruses have occurred in Australia and Southeast Asia sporadically since their discovery in the 1990s, with case fatality rates of up to 90%, including an outbreak of NiV in India starting in January 2026 [2,3,4,5]. Individuals who survive an acute HNV infection may still face long-term challenges due to the extent of neurological and respiratory damage caused by the virus [6]. Adding to these concerns, HNVs can infect a variety of animal reservoirs—bats, rodents, horses, and pigs—raising the possible risk of animal-to-human transmission (and vice versa) and by extension, pandemic potential [7,8,9]. Despite their rarity, the disease burden, high mortality, and wide species tropism exhibited by NiV and HeV have resulted in severe socioeconomic consequences in past outbreaks [7]. The 1999 NiV outbreak alone resulted in the culling of more than one million pigs and a consequential estimated total economic impact of USD 582 million [10]. Moreover, in addition to NiV and HeV, the more recent discovery of additional HNVs, such as Mojiang virus (MojV), Langya virus (LayV), Ghana Virus (GhV), and most recently Camp Hill Virus (CHV), further underscores the potential threat this genus poses [3,11,12,13,14,15,16]. LayV and MojV have both been associated with disease in humans, with LayV causing respiratory and febrile illness in 35 individuals in China in 2022, and MojV suspected to be responsible for the uniformly fatal respiratory disease of three miners in China in 2012 [12,17,18]. While the virulence of CHV and GhV in humans is unknown, their presence in North America and Africa (respectively)—outside of the typical regions of HNV infection—is concerning and emphasizes the need for more rigorous study of this genus of emerging viruses to develop broad preventative and treatment strategies [13,14].

Efforts are currently underway to develop prophylactics and therapeutics against HNVs, including the mRNA-1215 vaccine and the NiV- and HeV-neutralizing monoclonal antibody (mAb) m102.4 [19,20]. However, despite these efforts, no FDA-approved interventions exist. Further, none of the interventions currently in development target any HNVs beyond NiV or HeV [21]. Should NiV or HeV mutate, or an outbreak be caused by any other HNV, current therapeutics and prophylactics in development will be rendered totally ineffective. Given the highly pathogenic nature of HNVs, their wide range of animal hosts, and their potential for severe socioeconomic impact, there is an urgent need for effective and broad countermeasures to curb the spread and mortality associated with HNV outbreaks. At the time of writing, there are four Phase I clinical trials on clinicaltrials.gov for vaccines to prevent NiV and HeV infection (all completed), yet no vaccines or other countermeasures in development target any other HNVs [19,20,22,23,24]. Effective vaccines are a vital prophylactic tool against emerging viral pathogens and can prevent infection, death, and spread [25,26]. Given the diversity within the HNV genus and the wide species tropism, the development of broad, pan-genus vaccines is of the utmost importance.

Here, we explore sequential vaccination regimens against some of the most divergent HNVs to develop a pan-HNV vaccination strategy. To this end, we vaccinated rhesus macaques (RMs) with lipid nanoparticle-encapsulated mRNA vectors (mRNA-LNPs) encoding the attachment glycoprotein (gG) from NiV, GhV, and LayV, using two pan-HNV sequential heterologous regimens designed to maximize breadth (*n* = 2 per group), along with two additional animals receiving distinct regimens aimed at either homologous boosting (NiV-NiV-NiV) or targeted exposure to less well-characterized HNVs (GhV-LayV-GhV). The two pan-HNV sequential heterologous vaccination orders were chosen based on phylogenetic relationships [27,28]. In the first strategy (NiV-LayV-GhV), we prioritized vaccinating first against two of the most phylogenetically distant HNVs (NiV and LayV) before a final boost against a more phylogenetically central HNV, GhV. In the second strategy (GhV-NiV-LayV), we chose to prime with a phylogenetically central HNV, GhV, before boosting against more phylogenetically distant members of the genus (i.e., NiV and LayV). Together, this study suggests that sequential heterologous mRNA-LNP vaccination may be a promising strategy for eliciting cross-reactive Ab responses across the HNV genus.

## 2. Materials and Methods

### 2.1. Soluble Oligomeric gG Probe Design and Validation

Soluble oligomeric probes were generated from the attachment glycoprotein G (gG) ectodomains from five HNV members: NiV (AA71-602), HeV (AA71-602), GhV (AA87-632), MojV (AA66-625), and LayV (AA63-624). For each construct, the ectodomain of gG was cloned into a mammalian expression vector containing an N-terminal AviTag and 10XHis tag. Proteins were expressed in Expi293F and/or ExpiCHO cells (Gibco, Grand Island, NY, USA), purified by nickel-nitrilotriacetic acid (Ni-NTA) affinity chromatography, followed by buffer exchange using molecular weight-cutoff centrifugation columns (Millipore, Carlsbad, CA, USA). Size and oligomeric state were confirmed via SDS-PAGE and visualized using GelDoc imager (Bio-Rad, Hercules, CA, USA), with Coomassie staining under both reducing and non-reducing conditions.

Probe specificity was determined by indirect ELISA using known anti-HNV gG mAbs. NiV and HeV gG probes were detected with mAb m102.4 (produced in-house), GhV gG with 10C4-LY (absolute antibody, Newark, CA USA), MojV gG with 10G2-LY (absolute antibody, Newark, CA, USA), and LayV gG with the LayV/MojV cross-reactive mAb 6E5-LY (absolute antibody, Newark, CA, USA). A soluble form of SARS-CoV-2 Wuhan strain receptor-binding domain (RBD) was included as a negative control and detected using in-house mAb 22950_BS. High-binding plates (Corning, Glendale, AZ, USA) were coated with 5 µg/mL of each soluble oligomeric probe in bicarbonate coating buffer and incubated overnight at 4 °C. The next day, plates were washed four times with 1X PBS + 0.1% Tween-20 (PBST) and blocked using 1X PBS + 5% non-fat dry milk for 1 h at 37 °C. After blocking, plates were washed again, and primary mAbs were added at 10 µg/mL and incubated at 37 °C for 1 h. Plates were washed once more before adding a horseradish peroxidase (HRP)-conjugated secondary mAb, anti-human IgG-HRP, at a 1:10,000 dilution (SouthernBiotech, Birmingham, AL, USA). Finally, plates were washed and developed for 5 min using 3,3′,5,5′-tetramethylbenzidine (TMB) peroxidase substrate (Millipore, Carlsbad, CA, USA) before quenching with ELISA stop solution (SouthernBiotech, Birmingham, AL, USA) and reading absorbance at 450 nm on a Cytation 7 multimodal reader (BioTek, Winooski, VT, USA).

### 2.2. Research Animals and Ethics Statement

Six Indian RMs (*Macaca mulatta*) were used in this study. RMs were housed at the Wisconsin National Primate Center (WNPRC) at the University of Wisconsin-Madison and were cared for in compliance with the guidelines of the Weatherall report and the National Research Council’s Guide for the Use and Care of Laboratory Animals [29,30]. Experimental protocols were approved by the University of Wisconsin Graduate School Animal Care and Use Committee. Detailed descriptions of animal caretaking procedures (e.g., food, housing, enrichment activities, and standard medical care) can be found in the Research Animals and Ethics Statement in the Materials and Methods sections of our previous studies performed at the WNPRC [31,32,33]. During all procedures performed throughout this study, including vaccinations and blood draws, animals were anesthetized with ketamine (intramuscular, 5–12 mg/kg).

### 2.3. In Vitro Validation of Vaccine Constructs

mRNA constructs encoding full-length membrane-bound forms of gG from NiV, GhV, and LayV were codon-optimized for expression in mammalian cells and transcribed in vitro. The resulting mRNA was encapsulated in lipid nanoparticles (LNPs) for immunization. To validate expression, Expi293F cells were transfected at a density of 2-3e6 viable cells/mL with mRNA-LNPs encoding NiV, GhV, and LayV gG. Plasmid DNA encoding the same constructs was used as a positive control, and transfection complexation was performed using FectoPRO transfection reagent (Polyplus, Illkirch-Graffenstaden, France) and Opti-MEM reduced serum media (Gibco, Grand Island, NY, USA). Cells were incubated for 24 h at 37 °C with 5% CO_2_ and 200 rpm shaking speed before flow cytometric analysis. After 24 h, cells were washed with flow wash buffer (1X PBS + 1% FBS) and stained with a far-red fluorescent live/dead dye (Thermo Scientific, Waltham, MA, USA). Surface expression of NiV, GhV, or LayV gG was detected using primary mAb nAH1.3 (absolute antibody, Newark, CA, USA), 10D5-LY (absolute antibody, Newark, CA, USA), or 6E5-LY (absolute antibody, Newark, CA, USA), respectively, and anti-huIgG1-PE for NiV and LayV (polyclonal, Jackson ImmunoResearch Laboratories, West Grove, PA, USA) or anti-msIgG1-AF488 for GhV (clone HP6017, BioLegend, San Diego, CA, USA) secondary. Mock-transfected cells stained under identical conditions were used to establish gates for positive surface expression.

### 2.4. Rhesus Macaque Vaccination Regimens

Six Indian-origin RMs (*Macaca mulatta*) were enrolled under protocols approved by the Institutional Animal Care and Use Committee (IACUC) of the Wisconsin National Primate Research Center. Each animal was vaccinated intramuscularly in the hind leg with 100 µg of LNP-encapsulated mRNA encoding membrane-bound gG from NiV, GhV, or LayV. Animals were assigned to four distinct sequential vaccination regimens designed to explore both virus-specific and cross-reactive immune responses. Group 1 (*n* = 2) received mRNA-LNPs encoding NiV gG for the first dose, followed by LayV gG and GhV gG for the second and third doses, respectively. Group 2 (*n* = 2) received GhV gG, then NiV gG, and finally LayV gG. Group 3 (*n* = 1) received three sequential doses of NiV gG to model homologous boosting. Group 4 (*n* = 1) received GhV gG, followed by LayV gG, and then GhV gG again to evaluate responses against more divergent and less-characterized HNVs. Blood samples were collected prior to the first vaccination and weekly after each subsequent immunization. Plasma and peripheral blood mononuclear cells (PBMCs) were isolated from EDTA-anticoagulated blood by density gradient centrifugation with Ficoll-Paque Plus (Cytiva, Marlborough, MA, USA). PBMCs were washed in R10 medium—RPMI 1640 with GlutaMAX or L-glutamine (Gibco, Grand Island, NY, USA), 10% heat-inactivated fetal bovine serum (Thermo Scientific, Waltham, MA, USA), 1X antibiotic-antimycotic (Sigma-Aldrich, Burlington, MA, USA)—and red blood cells were lysed by resuspending PBMCs in ACK lysis buffer (Gibco, Grand Island, NY, USA) and incubating at room temperature for 5 min. Following an additional wash in R10 medium, PBMCs were counted, pelleted via centrifugation, and resuspended in cold cryopreservation medium—45% RPMI 1640 with GlutaMAX or L-glutamine, 45% heat-inactivated fetal bovine serum, and 10% DMSO (Sigma-Aldrich, Burlington, MA, USA). PBMCs were cooled to −80 °C at a controlled rate of 1 °C/min before being transferred to liquid nitrogen freezers for long-term storage. Serum was isolated by centrifugation in serum-separating blood collection tubes (BD Biosciences, San Jose, CA, USA) and stored at −80 °C until use.

### 2.5. Serum IgG Binding ELISA

To quantify serum IgG binding to each HNV gG antigen, high-binding 96-well plates (Corning, Glendale, AZ, USA) were coated with each soluble gG probe at 5 µg/mL in a bicarbonate coating buffer and incubated overnight at 4 °C. The following day, plates were washed four times with 1X PBST and blocked with 5% nonfat dry milk in 1X PBS for 1 h at 37 °C. Following an additional wash, serially diluted serum samples (1:10 to 1:40,960) were added and incubated for 1 h at 37 °C, followed by washing and detection with anti-human IgG-HRP at a 1:10,000 dilution. After a final wash, plates were developed using a TMB peroxidase substrate for 5 min at room temperature before quenching with ELISA stop solution. Absorbance was measured at 450 nm on a Cytation 7 multimodal reader. Antigen-specific binding responses were quantified as area under the curve (AUC) on a log10 scale using GraphPad Prism (version 10.6.1).

### 2.6. NiV, HeV, and GhV Pseudovirus Production

Lentiviral pseudoviruses encoding a firefly luciferase reporter gene were generated by co-transfecting HEK293T cells seeded at 60–80% confluency with the HIV-1 pNL4-3.luc.R^−^E^−^ backbone plasmid (NovoPro Bioscience, Shanghai, China) and plasmids encoding the attachment glycoprotein (gG) and fusion glycoprotein (gF) of the indicated virus. Plasmids were co-transfected at a mass ratio of (2:1):4 for (gG:gF):HIV using JetPRIME transfection reagent (Polyplus, Illkirch-Graffenstaden, France) at a DNA:JetPRIME ratio of 1:2.5 (µg:µL). Media was exchanged 4 h post-transfection, and viral supernatants were collected 48 h later for NiV and HeV, or 72 h later for GhV. Supernatants were clarified by filtration through a 0.22 µm membrane before storage.

For NiV pseudovirus, NiV gG and NiV gF plasmids were used. For HeV pseudovirus, HeV gG was paired with NiV gF, consistent with prior studies demonstrating improved infectivity of HeV pseudoviruses when combined with NiV gF while maintaining gG-dependent neutralization specificity [34]. For GhV pseudovirus, GhV gG was paired with a modified GhV gF construct previously described by Pernet et al., which improves fusogenicity and surface expression [35]. GhV pseudoviruses were produced in an OptiPRO serum-free medium supplemented with GlutaMAX (Gibco, Grand Island, NY, USA) and 1X antibiotic–antimycotic and concentrated approximately tenfold using Amicon Ultra 100 kDa centrifugal concentrators (Millipore, Carlsbad, CA, USA).

NiV and HeV pseudoviruses were aliquoted and stored at −80 °C until use. In contrast, GhV pseudovirus was stored at 4 °C to preserve infectivity, as even a single freeze–thaw cycle resulted in a substantial loss of signal and increased assay variability.

### 2.7. NiV, HeV, and GhV Pseudovirus Neutralization Assay

Serum samples were heat-inactivated at 56 °C for 30 min. Heat-inactivated sera were serially diluted, starting at 1:40, and incubated with pseudovirus for 1 h at 37 °C prior to infection of AD-293 cells (Agilent Technologies, Santa Clara, CA, USA) for NiV and HeV or MDCK cells (ATCC, Manassas, VA, USA) for GhV. After 48 h (NiV and HeV) or 72 h (GhV) of incubation, media were aspirated, cells were lysed, and luciferase activity was measured using the Bright-Glo luciferase assay system (Promega, Madison, WI, USA) on a Cytation 7 multimodal plate reader. NEUT_50_ titers were defined as the reciprocal dilution of serum that resulted in a 50% reduction in relative light units (RLU) compared to virus-only control wells and were calculated using non-linear regression in GraphPad Prism. NEUT_50_ values were set to a value of 1 for samples that exhibited no neutralization or that had calculated NEUT_50_ values below 1.

### 2.8. NiV, HeV, and GhV Receptor Blocking Assay

To assess the degree of neutralization by orthosteric receptor blocking, high-binding 96-well plates were coated overnight at 4 °C with NiV, HeV, or GhV gG at 1 µg/mL in bicarbonate coating buffer. The following day, plates were washed four times with 1X PBST before blocking with 1X PBS + 5% non-fat dry milk for 1 h at 37 °C. After blocking, plates were washed four times with 1X PBST once again before adding serially diluted heat-inactivated serum samples (titrated from 1:10 to 1:10,240) and incubating for 1 h at 37 °C. Following this incubation, plates were washed four times with 1X PBST once again before adding a soluble form of the host cell receptor, ephrin-B2 (sEFN-B2), at its EC80 concentration of 1 µg/mL in 1X PBS + 5% non-fat dry milk and incubating for 1 h at 37 °C. After the incubation, plates were washed four times with 1X PBST before adding streptavidin-HRP (SouthernBiotech, Birmingham, AL, USA) at a 1:6000 dilution and incubating for 1 h at 37 °C. After this final incubation, plates were washed four times with 1X PBST before developing for 5 min at room temperature with TMB peroxidase substrate, quenching with ELISA stop solution, and reading absorbance at 450 nm using the Cytation 7 multimodal reader. Percent inhibition was calculated relative to maximal receptor binding (no sera) wells, and ID_50_ values were defined as the dilution resulting in a 50% reduction in receptor binding and were calculated using non-linear regression in GraphPad Prism. ID_50_ values were set to a value of 1 for samples that exhibited no blocking or that had calculated ID_50_ values below 1.

## 3. Results

### 3.1. HNV gG Antigen Probes Exhibit Oligomeric Structure and Are Recognized by Published mAbs

**Figure 1:**
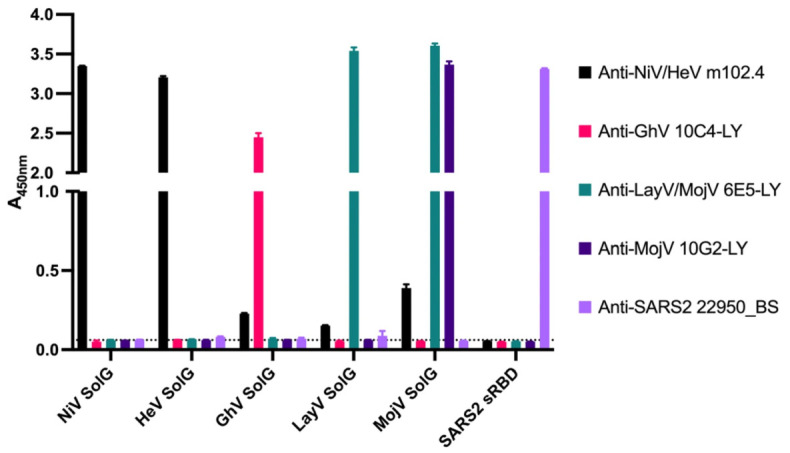
HNV soluble gG probes are bound by anti-HNV mAbs with high affinity and specificity. Binding was assessed by indirect ELISA with plates coated at 5 μg/mL with A_450 nm_ on the y-axis and indicated soluble gG probe on the x-axis. Primary antibodies indicated in legend were added at 10 μg/mL, and anti-huIgG-HRP secondary was used at 1:10,000 for all conditions. NiV and HeV soluble gG probes displayed strong binding by the anti-NiV/HeV gG mAb m102.4, while GhV and MojV gG probes displayed strong binding by anti-GhV and anti-MojV gG mAbs 10C4-LY and 10G2-LY, respectively. LayV gG, as well as MojV gG, displayed strong binding by the anti-LayV/MojV gG mAb 6E5-LY. Little to no cross-reactivity was observed between coating conditions for the same antibody. A soluble form of SARS-CoV-2 Wuhan RBD was used as a negative control, with detection by the in-house SARS-CoV-2 mAb 22950_BS. Bars represent mean A_450 nm_ ± SEM. Dashed horizontal line indicates the average of blank wells across all coating conditions.

To enable detection of HNV-specific Ab responses, we generated soluble oligomeric constructs of the attachment glycoprotein (gG) from NiV, HeV, GhV, MojV, and LayV. All gG constructs migrated at expected molecular weights by SDS-PAGE and exhibited a mixture of monomeric, dimeric, and tetrameric assembly in AKTA-FPLC size exclusion chromatography. We further evaluated each soluble oligomeric gG probe by indirect ELISA using published and/or commercially available mAbs (Figure 1). NiV and HeV gG probes were recognized by the anti-NiV/HeV cross-reactive mAb m102.4 (produced in-house), MojV gG by mAb 10G2-LY, GhV gG by mAb 10C4-LY, and LayV gG by the LayV/MojV cross-reactive mAb 6E5-LY [36,37]. All probes exhibited specific recognition by the expected published mAbs with minimal cross-reactivity between probes.

### 3.2. HNV gG Immunogens Are Efficiently Expressed Using mRNA-LNPs In Vitro

**Figure 2:**
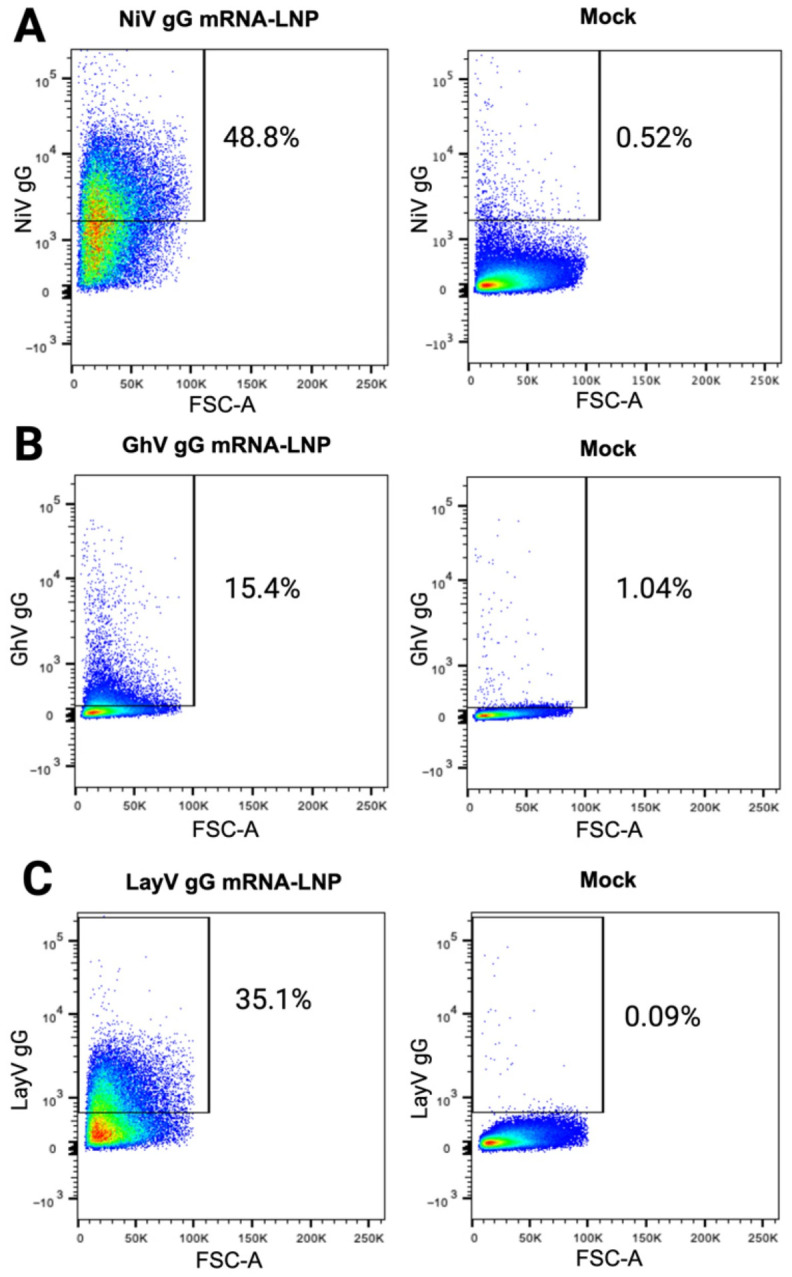
In vitro transfection with NiV gG-, GhV gG-, and LayV gG-encoding mRNA-LNPs yields surface-expressed gG. Expi293F cells were transfected with mRNA-LNPs encoding NiV gG (**A**), GhV gG (**B**), or LayV gG (**C**). At 24 h, cells were stained with a fluorescent viability dye and surface expression of NiV, GhV, or LayV gG was detected using primary mAb nAH1.3, 10D5-LY, or 6E5-LY, respectively, and anti-huIgG1-PE (for NiV and LayV) or anti-msIgG1-AF488 (for GhV) secondary. Gating is based on mock-transfected cells under identical staining conditions. Color scale represents event density, increasing from blue (low) to red (high). Figure panel labels were created using BioRender.com.

We first tested plasmids encoding membrane-anchored gG constructs of NiV, GhV, and LayV for their surface expression by transient transfection in Expi293F cells. Based on flow cytometric staining with virus-specific Abs, we selected the constructs that exhibited the best surface expression and produced them as lipid-encapsulated mRNA (mRNA-LNP) constructs. We then verified the in vitro expression of these mRNA-LNP constructs encoding membrane-anchored gG from NiV, GhV, and LayV in Expi293F cells by flow cytometry using virus-specific mAbs (Figure 2). All three mRNA-LNP constructs showed positive expression relative to the mock-transfected cells under the same staining conditions, though the detected surface expression of the GhV gG construct (15.4%) was markedly lower than that of NiV gG (48.8%) and LayV gG (35.1%).

### 3.3. HNV gG Vaccination Elicits Broad and Robust Binding Abs In Vivo in RMs

We selected six RMs from previous vaccination and challenge studies that had no documented HNV exposure and showed an undetectable baseline HNV gG binding by ELISA. Three of these RMs were previously infected with SIVmac239 but were all Mamu-B*08+ or Mamu-B*17+ elite controllers, maintaining low viral loads and exhibiting no opportunistic infections indicative of SIV-induced disease progression to AIDS [32,38,39]. Therefore, at the onset of the study, all six RMs were considered immunocompetent and HNV-naïve.

**Figure 3:**
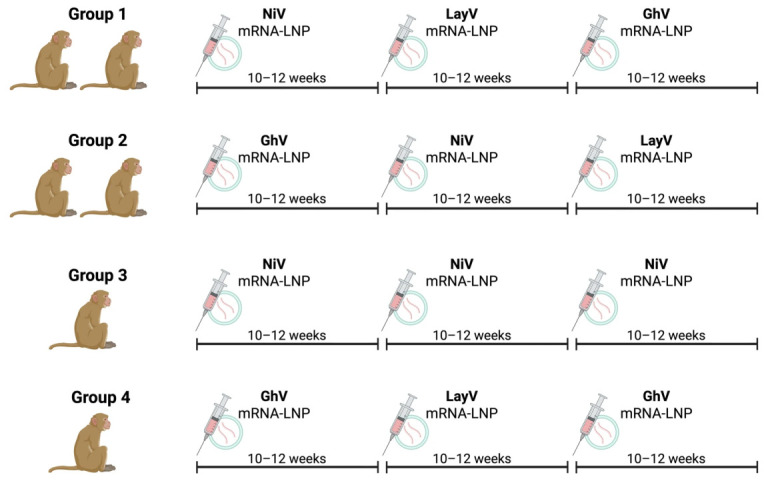
Vaccination strategy. Six RMs were sequentially vaccinated with mRNA-LNPs encoding for gG of NiV, GhV, and LayV in four distinct vaccination regimens. Vaccination order and timing are shown for each group (Group 1, *n* = 2; Group 2, *n* = 2; Group 3, *n* = 1; Group 4, *n* = 1). Graphic was created with BioRender.com.

**Figure 4:**
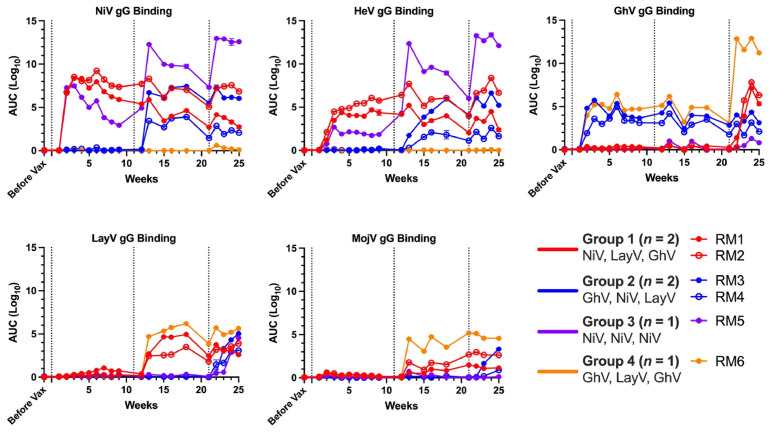
Sequential vaccination elicits cross-reactive serum binding Abs. Serum binding IgG responses by ELISA depicted as area under the curve (AUC) on a Log10 scale with time on the x-axis in weeks. Sera from vaccinated RMs were titrated serially from 1:10 to 1:40,960 and incubated with pre-blocked plates coated with each respective HNV gG at 5 μg/mL. Dashed vertical lines indicate timepoints for each vaccination. Lines are colored by vaccination group: Group 1 (*n* = 2) in red, Group 2 (*n* = 2) in blue, Group 3 (*n* = 1) in purple, and Group 4 (*n* = 1) in orange. Data points represent mean ± standard error in AUC estimate.

Next, we vaccinated these six RMs intramuscularly with 100 μg of each mRNA-LNP at three sequential timepoints, following one of four distinct regimens. In Group 1, two animals received mRNA-LNPs encoding NiV, LayV, and GhV in sequence to prioritize vaccination first against the most phylogenetically distant members of the genus. In Group 2, two RMs received the same antigens in a different order (GhV, NiV, LayV) to prioritize vaccination first against the most phylogenetically central member of the genus, before boosting against the most phylogenetically distant HNVs. In Group 3, a single RM received three sequential doses of NiV alone to model homologous boosting, while in Group 4, a single RM received a GhV-LayV-GhV regimen to examine immune responses to more divergent and less well-characterized HNVs (Figure 3). We next evaluated serum IgG binding responses by ELISA at serial timepoints following each vaccination (Figure 4). Both pan-genus vaccination regimens induced high-titer binding responses against all five HNVs with progressive increases in binding breadth following the second and third doses, with peak Ab binding log10 AUC values ranging from 3.9 to 9.2 against NiV, 2.2 to 7.7 against HeV, 5.0 to 6.3 against GhV, 3.1 to 5.0 against LayV, and 0.9 to 3.3 against MojV. In contrast, the NiV-only regimen yielded strong binding responses to NiV (peak AUC = 13.0) and HeV (peak AUC = 13.3), but failed to elicit detectable binding to GhV, LayV, or MojV. Conversely, the GhV-LayV-GhV regimen induced robust binding to GhV (peak AUC = 12.8), LayV (peak AUC = 6.2), and MojV (peak AUC = 5.2), but no cross-reactivity to NiV or HeV.

### 3.4. NiV, HeV, and GhV Pseudovirus Systems Are Neutralized by Known Neutralizing mAbs and Soluble Form of Host Cell Receptor

**Figure 5:**
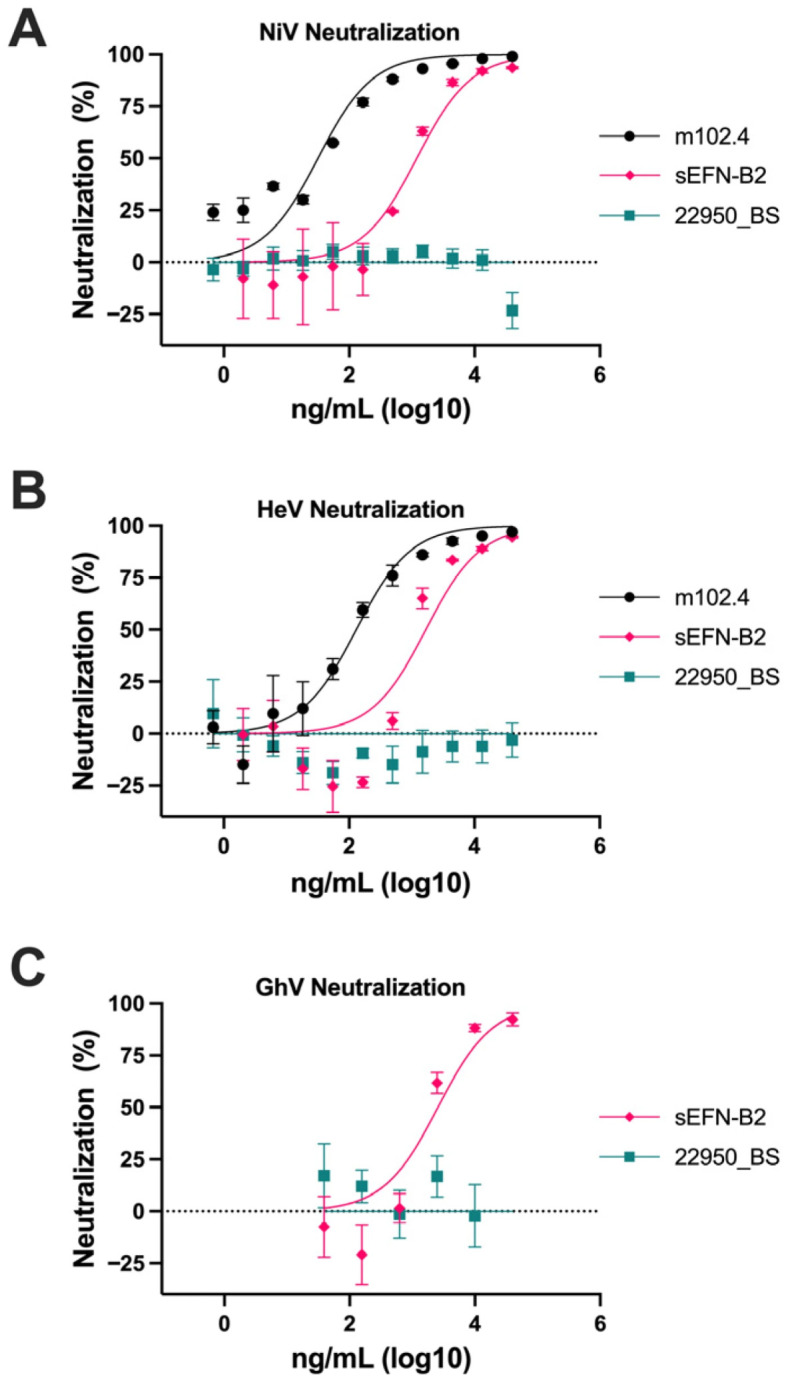
NiV, HeV, and GhV pseudovirus systems show neutralization by known neutralizing mAbs and soluble host cell receptor. Neutralization of (**A**) NiV, (**B**) HeV, or (**C**) GhV pseudovirus is depicted on the y-axis with concentration in ng/mL on a log10 scale on the x-axis. Black represents the NiV and HeV neutralizing mAb m102.4, pink represents the soluble form of host cell receptor ephrin-B2 (sEFN-B2), and teal represents the non-HNV-reactive negative control mAb 22950_BS. Data points represent mean ± SEM.

Next, we sought to validate our NiV, HeV, and GhV pseudovirus systems by neutralization assays with known neutralizing mAbs and/or a soluble form of their shared host cell receptor, ephrin-B2 (sEFN-B2) (Figure 5). The cross-reactive NiV/HeV-neutralizing mAb m102.4 neutralized NiV and HeV at concentrations consistent with previously reported NEUT_50_ values [40]. sEFN-B2 neutralized NiV, HeV, and GhV pseudoviruses in a dose-dependent manner, confirming that viral entry in each system is mediated by the canonical host cell receptor, ephrin-B2.

### 3.5. HNV gG Vaccination Elicits NiV-, HeV-, and GhV-Neutralizing Abs

**Figure 6:**
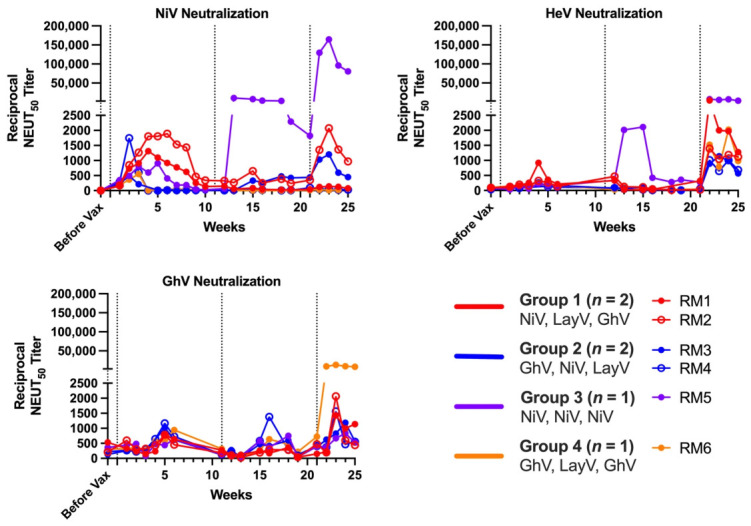
Sequential vaccination elicits potent serum neutralizing Ab responses against NiV, HeV, and GhV pseudoviruses. Reciprocal NEUT_50_ titer against NiV, HeV, or GhV pseudovirus is depicted on the y-axis with time on the x-axis in weeks. Dashed vertical lines indicate timepoints for each sequential vaccination. Lines are colored by vaccination regimen: Group 1 (*n* = 2) in red, Group 2 (*n* = 2) in blue, Group 3 (*n* = 1) in purple, and Group 4 (*n* = 1) in orange.

To assess functional Ab responses, we performed longitudinal serum neutralization assays using lentiviral particles pseudotyped with NiV, HeV, and GhV gG and gF glycoproteins (Figure 6). Animals in both heterologous pan-genus vaccination groups developed moderate neutralizing activity against NiV, HeV, and GhV with peak NiV NEUT_50_ titers ranging from: 1:1000 to 1:10,000, peak HeV NEUT_50_ titers ranging from 1:1000 to 1:4000, and peak GhV NEUT_50_ titers ranging from 1:1000 to 1:2000. The highest neutralization titer was observed in the NiV-only animal, which reached a NiV NEUT_50_ titer of 1:160,000 and a peak HeV NEUT_50_ titer of 1:7400, while showing limited cross-neutralization of more distant HNVs, with a peak GhV NEUT_50_ titer of 1:800. The GhV-LayV-GhV regimen elicited potent neutralizing responses against GhV, with a peak NEUT_50_ titer of 1:13,000, while showing more limited cross-neutralization of NiV and HeV, with peak NEUT_50_ titers of 1:100 and 1:2000, respectively.

### 3.6. HNV gG Vaccination Elicits Receptor-Blocking Abs

**Figure 7:**
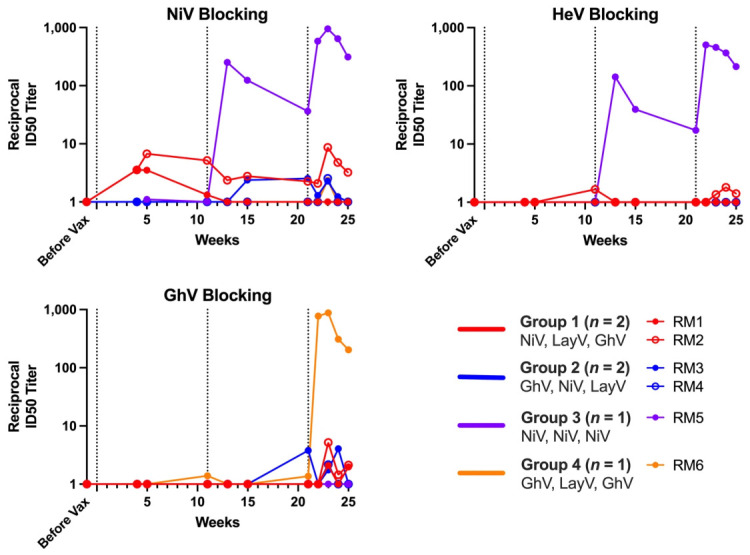
Sequential vaccination elicits Abs that block HNV binding to the EFN-B2 receptor. Reciprocal ID_50_ sEFN-B2 blocking titer against NiV, HeV, or GhV gG is depicted on the y-axis on a Log10 scale with time on the x-axis in weeks. Serially diluted sera samples (titrated from 1:10 to 1:10,240) were added to pre-blocked plates coated with each indicated HNV gG at 1 μg/mL and incubated for 1 h at 37 °C before the addition of biotinylated sEFN-B2 at its EC80 (1 μg/mL). Detection was performed in all conditions with streptavidin-HRP. Percent inhibition was calculated relative to maximal receptor binding (no sera) wells. Dashed vertical lines indicate timepoints for each sequential vaccination. Lines are colored by group: Group 1 (*n* = 2) in red, Group 2 (*n* = 2) in blue, Group 3 (*n* = 1) in purple, and Group 4 (*n* = 1) in orange.

To complement pseudovirus neutralization assays, we next assessed the ability of vaccine-induced Abs to block binding of NiV, HeV, and GhV gG to a soluble form of their host cell receptor, sEFN-B2, by competition ELISA (Figure 7). Across the two pan-genus heterologous vaccination groups, RMs mounted modest receptor blocking with peak half-maximal inhibitory serum dilutions (ID_50_) of less than 1:10. The NiV-only regimen elicited peak ID_50_ values of approximately 1:1000 and 1:500 against NiV and HeV, respectively, with no detectable receptor blocking against GhV. Conversely, the GhV-LayV-GhV regimen elicited potent receptor blocking against GhV (ID_50_ = 1:900), with limited blocking detected against NiV or HeV.

## 4. Discussion

HNVs remain among the most concerning emerging zoonotic pathogens due to their extreme case fatality rates, broad host range, and lack of licensed human vaccines or antivirals. The sporadic yet recurrent outbreaks of NiV and HeV, combined with the recent identification of additional and more phylogenetically distant HNVs, including GhV, LayV, MojV, and CHV, underscore the pandemic threat posed by this genus. Indeed, the danger posed by this genus is further emphasized by the 2026 NiV outbreak in India [5]. Importantly, the antigenic diversity among these viruses presents a major obstacle to vaccine development. A successful pan-HNV vaccine must elicit Abs capable of neutralizing both NiV and HeV, as well as more divergent viruses like GhV, LayV, and MojV.

The recent discovery of CHV, a novel HNV isolated from rodents in Alabama, underscores the growing geographic and phylogenetic diversity of the HNV genus [15,16]. CHV represents the first documented HNV in North America, raising concerns about zoonotic potential outside the historically endemic regions of Southeast Asia and Australia. While our study focused on five known HNVs, future vaccine strategies will need to account for the expanding antigenic landscape, including newly identified members like CHV. This diversity emphasizes the importance of developing vaccination approaches that prioritize cross-reactivity and functional breadth. Against this backdrop, our study sought to evaluate sequential heterologous mRNA vaccination as a strategy to induce broad Ab responses spanning these divergent lineages.

Herein, we evaluated the strength and breadth of Ab responses elicited by sequential heterologous mRNA-LNP vaccination regimens in RMs targeting gGs from divergent HNVs in a pilot study. Our results, though preliminary in nature, demonstrate that heterologous vaccination strategies can elicit broad binding Ab responses in nonhuman primates against five major members of the HNV genus—NiV, HeV, GhV, MojV, and LayV—while also achieving moderate neutralization of NiV, HeV, and GhV lentiviral pseudoviruses. These findings support the feasibility of pan-HNV vaccine design using heterologous sequential immunization with diverse HNV gG antigens.

Though our small sample sizes limit the ability to perform statistical analyses and form definitive conclusions, we found that the two pan-genus heterologous regimens (Group 1: NiV-LayV-GhV; Group 2: GhV-NiV-LayV) induced serum binding Abs to all five HNV gG antigens, highlighting the potential of sequential exposure to distinct antigens in driving breadth. These broad Ab binding responses were accompanied by moderately potent NiV-, HeV-, and GhV-neutralizing activity (peak NEUT_50_ titers between 1:1000 and 1:10,000). Importantly, the log10 IgG binding AUC values and NEUT_50_ titers observed herein are on par with or better than those seen in either previous HNV vaccination studies or Moderna’s homologous prime-boost mRNA-1273 COVID-19 experiments in RMs [41,42,43]. However, these neutralizing Ab responses paled in comparison to the homologous NiV-only regimen (Group 3), which produced a potent but narrow response against NiV and HeV (peak NiV NEUT_50_ = 1:160,000, peak HeV NEUT_50_ = 1:7400), with limited cross-reactive binding or neutralizing Ab responses against more distant HNVs. Conversely, Group 4, which received the GhV-LayV-GhV regimen, mounted robust binding Ab responses to GhV, LayV, and MojV and potent GhV neutralization (peak NEUT_50_ = 1:13,000) but showed limited binding or neutralization of NiV beyond baseline, further underscoring the phylogenetic separation of GhV- and LayV-like viruses from NiV and HeV. Somewhat surprisingly, this animal developed modest neutralizing Ab responses against HeV, with a peak NEUT_50_ of 1:2000. This may reflect more antigenic similarity in shared neutralizing epitopes on GhV and HeV that are dissimilar on NiV gG, though NiV and HeV gG share much more sequence identity overall [44]. Collectively, these data highlight the immunological distance between members of the HNV genus and the need to include more diverse antigenic representatives in any pan-HNV vaccination strategy. Further, these findings underscore the balance between potency and breadth: homologous vaccination produces very high titers against closely related viruses but little coverage of distant lineages, whereas heterologous regimens elicit cross-reactive responses that come at the cost of maximal neutralization titers [45,46,47,48,49]. Resolving this trade-off will be central to optimizing pan-HNV vaccine strategies.

To investigate whether the vaccine-induced neutralization activity was mediated primarily by Abs targeting the receptor-binding site (RBS), we next assessed the inhibition of sEFN-B2 binding to NiV, HeV, and GhV gG by vaccinee serum in a competition ELISA. This assay captures the contribution of the subset of neutralizing Abs that function by direct inhibition of gG-mediated receptor engagement as opposed to allosteric inhibition of viral entry. Both pan-genus heterologous vaccination regimens elicited weak sEFN-B2 blocking, with peak ID_50_ titers of less than 1:10 against NiV, HeV and GhV. In contrast, the NiV-only regimen elicited appreciable sEFN-B2 blocking titers against NiV and HeV only after the second vaccination and showed no blocking above baseline against GhV. Notably, the sEFN-B2 blocking titers against NiV and HeV in this regimen matched much more closely than the NiV- and HeV-neutralizing titers, indicating that cross-reactive neutralization of HeV is likely driven by RBS-mediated neutralization. Similarly, the GhV-LayV-GhV regimen only elicited sEFN-B2 blocking against GhV after the third vaccination (i.e., second exposure to GhV) and showed no sEFN-B2 blocking above baseline against NiV and HeV. Together, these findings detail the limited RBS-directed neutralizing Ab responses elicited by heterologous vaccination and demonstrate that multiple exposures against the same antigen may be required for driving RBS-directed neutralization. Heterologous regimens appear to broaden responses, perhaps through eliciting Abs targeting conserved allosteric neutralizing epitopes, though more work is needed to fully resolve the epitopes preferentially targeted by these regimens. Given the evolutionary constraints placed on the RBS of ephrin-B2-utilizing HNVs, prioritization of RBS-specific Ab responses may be advantageous in mitigating escape [50].

One of the most significant advances in this work is the establishment of a functional GhV pseudovirus platform for assessing Ab-mediated neutralization. While prior studies have inferred neutralization by assessing the inhibition of GhV gG-mediated ephrin-B2 binding, functional neutralization of GhV entry has not previously been demonstrated [51,52]. By incorporating a modified GhV gF construct described by Pernet et al. and optimizing production conditions to preserve infectivity, we generated a robust and reproducible GhV pseudovirus suitable for BSL-2-level evaluation of Ab function [35]. Further, because no authentic GhV isolates currently exist, this pseudovirus system represents the only available means of assessing GhV-specific neutralization. Importantly, our platform was validated both mechanistically and functionally: (i) neutralization titers correlated with inhibition of sEFN-B2 binding in receptor-blocking ELISAs, and (ii) sEFN-B2 itself neutralized GhV pseudovirus in a dose-dependent manner, confirming that viral entry is ephrin-B2-dependent. While receptor-blocking assays serve as a useful proxy for functional neutralization, true pseudovirus neutralization provides a more comprehensive measure of Ab efficacy, as it also captures both allosteric and orthosteric mechanisms of viral inhibition. Indeed, we showed that serum neutralizing Ab titers against pseudovirus can reach much higher levels than those captured by orthosteric receptor blocking alone. Together, these findings establish the first validated pseudovirus system for measuring GhV neutralization and provide a practical framework for evaluating vaccines and mAbs against other divergent HNVs for which live viral isolates are unavailable.

One major limitation of this study is that our current analysis is limited to the neutralization of NiV, HeV, and GhV pseudoviruses, and it remains unclear whether the broad binding responses elicited by heterologous vaccination regimens translate into functional neutralization of MojV and LayV. Planned studies using pseudoviruses bearing LayV and MojV glycoproteins should help determine whether these regimens can induce meaningful cross-neutralization beyond NiV, HeV, and GhV, though no groups have yet published functional pseudovirus systems for LayV or MojV, to our knowledge. Further, the use of pseudoviruses, though an improvement over receptor blocking alone, does not perfectly represent true neutralization against an authentic live virus. Though no viral isolates of GhV, LayV, or MojV have successfully been isolated and passaged, we plan on performing serum neutralization assays against authentic live NiV and HeV in a BSL-4 setting in future experiments. Additionally, the neutralizing and receptor-blocking titers declined within weeks of the final vaccination. Though some waning in serum Ab titers is expected, more work is needed to fully resolve the durability of immune responses elicited by these vaccine regimens. Beyond this, small animal (e.g., ferret) or non-human primate challenge studies with authentic live virus are needed to better resolve the in vivo efficacy of these sequential pan-HNV vaccination strategies [43]. To further investigate the nature of the cross-reactive Ab response, we may also perform longitudinal clonal analysis of memory B cells using single-cell RNA sequencing. This approach could allow us to resolve the V(D)J gene usage and sequence profiles of rare B cells that exhibit broad or pan-genus binding. Isolated mAbs from these lineages could then be tested against known and emerging HNVs, including CHV and Angavokely virus (AngV), to evaluate their breadth and neutralization potential. These efforts may ultimately guide the design of next-generation immunogens through reverse vaccinology [53].

Another important consideration is the small number of animals per group (*n* = 1–2), which limits statistical power and precludes broader generalization. Despite this, the internal consistency observed within regimens—especially the contrasting breadth profiles between groups—suggests that the differences observed are biologically meaningful. Indeed, while inter-animal variability was evident within Group 1 and within Group 2, especially for neutralization and receptor blocking titers, these differences were modest in magnitude, with peak responses differing by no more than threefold. Nevertheless, more work with larger sample sizes is needed to confirm these interpretations. Moreover, this study was designed as a proof-of-concept pilot study to evaluate the immunogenicity of sequential mRNA-LNP vaccination and identify promising strategies for downstream B cell repertoire analysis.

In future studies, we also plan to assess serum reactivity against more phylogenetically distant and newly identified members of the HNV genus, including CHV and AngV, to determine whether the breadth observed here extends beyond the five viruses evaluated. We selected NiV, HeV, GhV, LayV, and MojV based on their confirmed or suspected association with human disease and/or their official classification within the genus at the time of vaccine design [11,12,54]. Viruses such as AngV and CHV, which had not yet been formally recognized or linked to human pathogenicity, were excluded from the initial panel. Demonstrating binding, or ideally neutralization, against these more divergent viruses would provide compelling support for the utility of sequential vaccination in achieving pan-genus protection through the development of rare cross-reactive B cells, in contrast to single-dose multivalent approaches. Additionally, these rare cross-reactive B cells could feasibly be sorted using our soluble oligomeric HNV gG probes and sequenced for the isolation and development of novel, broadly reactive neutralizing mAbs against this deadly and diverse genus of viruses [55,56]. Given the exceptionally limited number of surviving individuals exposed to HNVs, banked PBMC samples from these sequentially vaccinated RMs could prove to be a highly useful resource in HNV-targeting mAb discovery efforts.

Lastly, while our current study focused on Ab responses, future work may also consider whether T cell-mediated immunity contributes to the magnitude or durability of vaccine-elicited protection. Though Abs represent a first line of defense for the adaptive immune system, the role of T cells in mediating vaccine response, efficacy, and potential undesirable side effects should not be overlooked [45,57,58]. gG-based vaccines can induce both potent neutralizing Ab responses and gG-specific T cell responses; however, they are restricted to presenting T cell epitopes within gG. As such, they may fail to elicit T cell responses against other viral antigens, which could broaden protective immunity across divergent HNVs. Understanding the full body of immune mechanisms involved will be critical as we move toward the development of durable, broadly protective countermeasures against future HNV outbreaks.

In conclusion, our findings provide foundational evidence that sequential mRNA-LNP vaccination using divergent gG immunogens is a viable approach for eliciting cross-reactive Ab responses across the HNV genus. While the small sample sizes limit our ability to form statistically meaningful conclusions and challenges remain in achieving both potency and breadth, this work lays important groundwork for optimizing pan-HNV vaccine strategies.

## Data Availability

The raw data supporting the conclusions of this article will be made available by the authors on request.

## Abbreviations

The following abbreviations are used in this manuscript:

HNV	Henipavirus
NiV	Nipah virus
HeV	Hendra virus
GhV	Ghana virus
LayV	Langya virus
MojV	Mojiang virus
CHV	Camp Hill virus
AngV	Angavokely virus
Ab	Antibody
gG	Attachment glycoprotein G
gF	Fusion glycoprotein F
LNP	Lipid nanoparticle
RBS	Receptor-binding site
PBMC	Peripheral blood mononuclear cell
